# Systematic Review and Meta-analysis of Linkage to HIV Care Interventions in the United States, Canada, and Ukraine (2010–2021)

**DOI:** 10.1007/s10461-023-04121-0

**Published:** 2023-07-31

**Authors:** Julie H. Levison, Paola Del Cueto, Jaime Vladimir Mendoza, Dina Ashour, Melis Lydston, Kenneth A. Freedberg, Fatma M. Shebl

**Affiliations:** 1 Division of General Internal Medicine, Department of Medicine, Massachusetts General Hospital, 100 Cambridge Street, Suite 1600, Boston, MA 02114 USA; 2 Division of Infectious Diseases, Department of Medicine, Massachusetts General Hospital, Boston, MA USA; 3 Medical Practice Evaluation Center, Massachusetts General Hospital, Boston, MA USA; 4 Treadwell Library, Massachusetts General Hospital, Boston, MA USA; 5 Harvard Medical School, Boston, MA USA

**Keywords:** Linkage to care, Engagement in care, HIV/AIDS, Systematic review, Meta-analysis

## Abstract

We conducted a systematic review and meta-analysis of interventions targeting linkage to HIV care in the US, Canada, and Europe. We searched six databases (PubMed, Embase, Cochrane Library, Web of Science and CINAHL). Inclusion criteria were English language studies in adults in the US, Canada**,** or Europe, published January 1, 2010 to January 1, 2021. We synthesized interventions by type and linkage to care outcome. The outcome was cumulative incidence of 3-month linkage. We estimated cumulative incidence ratios of linkage with 95% confidence intervals (CIs). We screened 945 studies; 13 met selection criteria (n = 1 from Canada, n = 1 from Ukraine, n = 11 from the US) and were included after full text review (total 37,549 individuals). The cumulative incidence of 3-month linkage in the intervention group was 0.82 (95% CI 0.68–0.94) and control group 0.71 (95% CI 0.50–0.90); cIR of linkage for intervention versus control was 1.30 (95% CI 1.13, 1.49). Interventions to improve linkage to care after HIV diagnosis warrant further attention.

**Supplementary Information:**

The online version contains supplementary material available at 10.1007/s10461-023-04121-0.

## Introduction

Early linkage to care and timely access to antiretroviral therapy (ART) are key for effective immunological response, reduction in HIV-related mortality, as well as averting HIV transmission [1–3]. The UNAIDS goals prioritize that by 2030, 95% of people with HIV (PWH) on HIV treatment should achieve viral suppression, a target consistent with the US National HIV/AIDS Strategy [4, 5]. Yet in order to achieve viral suppression an individual with HIV must not only test and receive the HIV diagnosis (HIV testing), but link to HIV care (linkage to care) and receive antiretroviral therapy (ART receipt).

The World Health Organization defines linkage to care as the time from HIV diagnosis to enrollment in HIV care [6]. Linkage to care is a conduit to both HIV primary care services, importantly ART, and non-clinical services including food, housing, legal support, and substance use and mental health care. However, in the US, only 80% of persons with HIV are linked to care within one month of diagnosis; this is lowest for Blacks/African Americans and American Indians/Alaskan Natives at 77%, compared with non-Hispanic whites at 83% and Hispanics at 82%. Linkage to care within one month of diagnosis in people who inject drugs is 76% and for individuals 13 to 24 years old is 77%, both lower than the overall linkage in PWH of 80% [7]. An HIV surveillance study from Ontario, Canada examined linkage to care within one month of HIV diagnosis and found that 65.3% of persons with HIV were linked to care in 2018, with more males than females linked within one-month [8]. A meta-analysis and systematic review in Europe found that of all new HIV diagnoses, approximately 71% were linked to care after diagnosis, though the time interval was not specified [9]. Meta-analyses from the US and Europe demonstrate that factors associated with lower rates of linkage to care were Black race, heterosexual and injection drug use as modes of transmission, younger age at diagnosis, lower socioeconomic status, feeling well at diagnosis, and diagnosis in a facility without co-located HIV treatment and primary care services [10].

Current evidence-based literature and expert recommendations for timely linkage to care emphasize the role of case management (CM) [11, 12]. Trained social workers and care coordinators could help individuals newly diagnosed with HIV identify their internal strengths to translate to successful linkage to care. Additional studies have identified co-location of HIV testing services with HIV care and other medical specialties and behavioral health [13], orientation to the HIV clinic [14], and opt-out HIV testing in routine clinical settings [15] as effective strategies to improve linkage to HIV care. Questions remain, however, as to how best to tailor interventions to those most at risk for delayed linkage to HIV care and which kind of interventions provide incremental benefit over others and warrant further evaluation or dissemination. Countries differ in the heterogeneity of their populations and contextual circumstances, which may necessitate adaptation of linkage to care interventions. Furthermore, different regions vary in their HIV profiles and targets for progress in the HIV care continuum [16]. Our objective was to systematically review the literature to understand the effect of diverse types of linkage to care interventions in the US**,** Canada, and Europe, with a focus on Ukraine where the HIV literature has predominated on linkage to care.

## Methods

### Design

This review followed PRISMA 2020 guidelines [17]. Eligible studies were in English, conducted in Europe, Canada and the US, published between January 1, 2010 and January 1, 2021, and included subjects age 18 years and older. Studies needed to report a defined quantitative outcome of linkage to care. We included experimental and non-experimental study designs and excluded studies that were modeling, qualitative, reviews and commentaries, and those in which the authorship team (JHL, PDC, KAF, FMS) deemed by consensus were not focused on linkage to care interventions, such as studies that focused on HIV testing interventions. This study was exempt from human subjects review as the analysis relied on synthesis of de-identified published outcome data.

### Information Sources

Electronic searches for published literature were conducted by a medical librarian (ML) using Embase.com, Web of Science, CINAHL (Cumulative Index to Nursing and Allied Health Literature) Complete, MEDLINE via PubMed, and Cochrane Central Register of Controlled Trials via Ovid and Cochrane Database of Systematic Reviews via Ovid. All of the database searches except MEDLINE were run in January 2021. The MEDLINE search was run in February 2022 to fix a syntax error and pre-dated to match the rest of the searches.

### Search Strategy

The search strategy incorporated controlled vocabulary and free-text synonyms for the concepts of linkage to care, HIV, and intervention studies (Online Appendix 1). No restrictions on language or any other search filters were applied. All identified studies were combined and de-duplicated in a single reference manager (EndNote) and then uploaded into Covidence systematic review management software [18].

### Selection Process

Search results were imported to Covidence software for initial review. Two researchers (JHL and PDC) independently evaluated titles, then performed a review of abstracts to identify potentially relevant studies. They each conducted a full-text review according to established inclusion and exclusion criteria, with a third reviewer (FMS) resolving disagreements.

### Data Collection Process

From eligible included studies we extracted data using a uniform data entry sheet maintained on Covidence software. Three researchers (JHL, PDC, JVM) independently abstracted the data from eligible studies. In the presence of discrepancies, a fourth reviewer (FMS) resolved the disagreements.

### Data Items

For each study, we extracted data regarding intervention characteristics and types of interventions evaluated for both intervention and comparator groups. We collected study characteristics including authors, title, publication year, study location and setting, study design, objectives, and recruitment method. For subject characteristics, we collected the sample size, target population, study attrition, and potential outcome predictors when available*,* including age; sex or gender, grouped as male, female, or transgender; race/ethnicity, grouped as Black, White, Other, or Hispanic; socioeconomic status grouped as employed and unemployed; sexual orientation grouped as men who have sex with men and heterosexual; education grouped as less than a high school education and high school graduate and higher; and substance use grouped as ever and no injection drug use. Study outcome measures related to linkage to care and outcome order were extracted as reported in the article.

### Study Risk of Bias Assessment

We used two risk of bias assessment tools (Supplementary Materials, Additional Methods). For randomized trials we used the revised Cochrane “Risk of bias” tool for randomized trials (RoB 2.0) [19]. For non-randomized studies we used the “Risk of bias” tool called ROBINS-I [20]. Two reviewers (JVM and DA) independently evaluated all of the studies. In the presence of discrepancies, a third reviewer (FMS or JHL) resolved the disagreements. We used Risk-of-bias VISualization (robvis) to create risk-of-bias plots [21].

### Synthesis Methods

#### Interventions

We categorized linkage to care interventions into five broad groups: 1) CM; 2) community health worker (CHW); 3) healthcare provider; 4) structural, and 5) multiple component (i.e. interventions that included more than one intervention modality).

#### Outcome

Studies did not report linkage to care outcomes in a standardized fashion. Since most studies reported linkage to care within three months, we used the reported cumulative proportions with the time frame, or the number of events and the time frame to estimate linkage rate per month, and subsequently estimated the 3-month linkage to care probability (cumulative incidence) from the calculated linkage rate (Supplementary Materials). The main outcome was 3-month linkage to care cumulative incidence.

We planned to use the cumulative incidence and the cumulative incidence ratios (cIR) of the outcome reported by each study. However, studies often did not provide measures of cIR, but provided other estimates such as time to linkage, or the frequencies of the outcome in the intervention and the comparator groups. Therefore, we estimated a pooled cIR using two approaches; (1) calculating the estimate from all studies (single-arm and 2-arm studies) using a meta-regression approach, and (2) restricting to studies that included a comparator group (Supplementary Materials).

Meta-analysis was conducted using MetaXL 5.3 (EpiGear International, Sunrise Beach, Australia www.epigear.com) and SAS 9.4 (Cary, NC, USA). We used double arcsine transformation to stabilize the variance and avoid confidence intervals (CIs) that fall outside the 0–1 range [22]. We used the method of inverse variance heterogeneity to weight the studies presented using forest plots [23]. We assessed statistical heterogeneity using the I^2^ statistic and Cochran’s Q χ^2^ test for heterogeneity and conducted subgroup analyses to explore the potential sources of heterogeneity [24–26]. To include single-arm studies we employed fixed-effect meta-regression in SAS, using the transformed effect size (double arcsine transformation) and the inverse variance heterogeneity weights calculated by MetaXL. To address inclusion of heterogeneous studies and overcome the underestimation of statistical errors usually encountered in random effects models, we used the inverse variance heterogeneity model, a fixed effect model with a quasi-likelihood-based variance structure that allows extraneous variation using an overdispersion correction. This method is a replacement of the standard random effects and fixed effect models to address the problems of both approaches [23, 27]. To obtain the correct error estimates, we estimated Huber–White robust standard errors [28, 29].

### Reporting Bias Assessment (Publication Bias)

We used Doi plot and the Luis Furuya-Kanamori (LFK) index, a quantitative indicator of Doi plot asymmetry, for the detection of publication bias [30].

## Results

### Study Selection

**Fig. 1:**
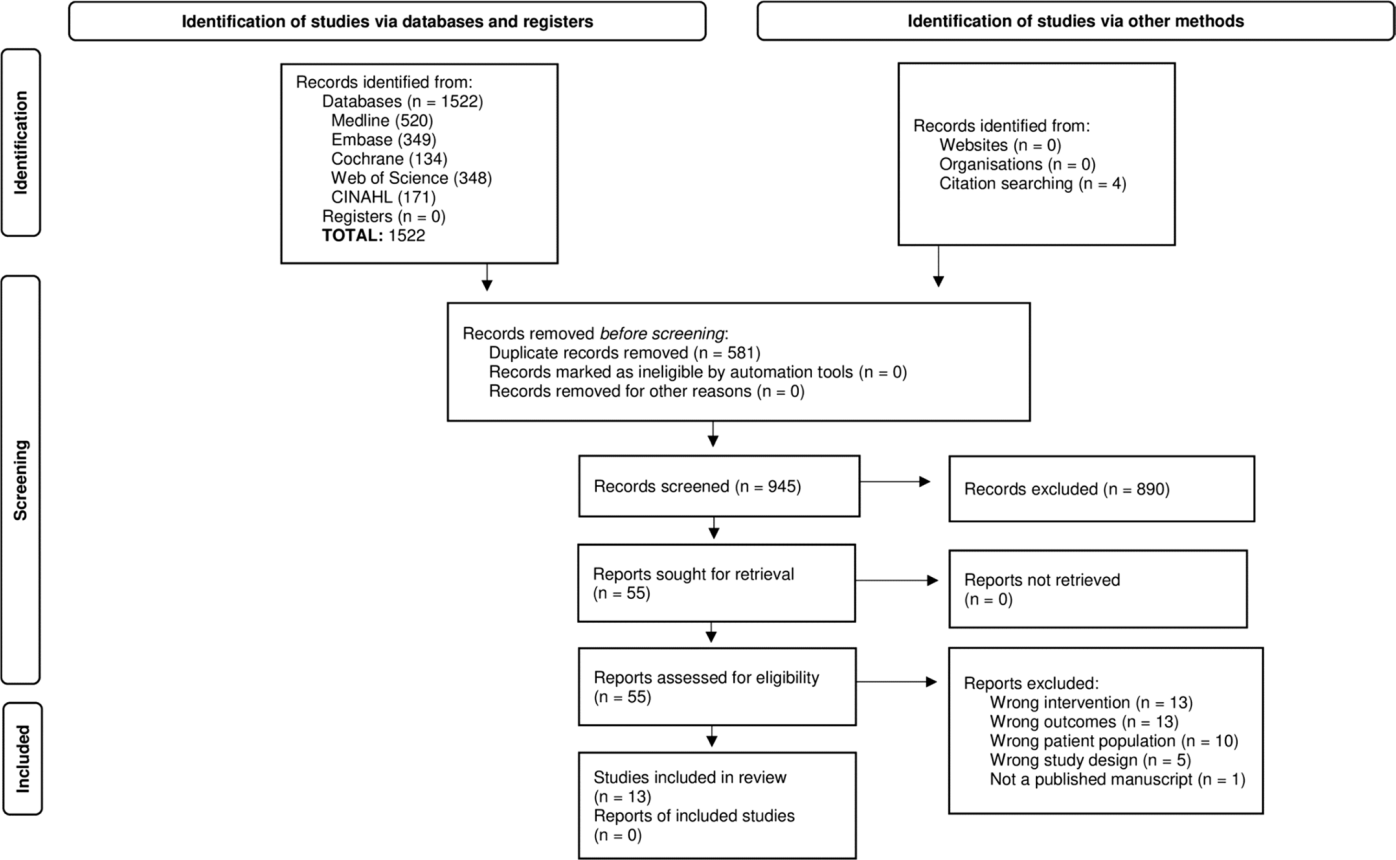
PRISMA 2020 flow diagram for new systematic reviews which included searches of databases, registers, and other sources [17]

We identified 1522 references by electronic database search that matched with relevant key terms and an additional four references by citation searching. After removing duplicates, we screened 945 records by the titles and abstracts to assess whether the references met the inclusion criteria, which yielded 55 records. We excluded 42 studies because they focused on either the wrong intervention, outcomes, patient population, or study design; one study was unpublished. The final sample included 13 studies. The total sample size was 37,549 people with newly diagnosed HIV (Fig. 1).

### Study Characteristics

We summarize key characteristics of included studies in Table 1. Four studies (31%) were quasi-experimental (pre-post studies) [31–34]; three (23%) were experimental studies, including one non-randomized experimental study [35] and two controlled trials [36, 37]. Six (46%) were cohort studies [38–43].

**Table 1:** Included linkage to care studies for a meta-analysis review of interventions to improve linkage to care in the United States, Canada, and Ukraine, 2010–2021

Study	Intervention type	Intervention description	Comparator	Target population	Sample size	Outcome	Intervention results	Comparator results		Significant results
*Quasi-experimental studies (pre–post)*										
Anderson et al. (2020) [32]	MC	Data-to-care and care coordination (2013–2015)	Care-as-usual services through community-based HIV organizations (2011–2013)	PWH, newly diagnosed and not in care in Louisiana, USA	N = 843 (T) N = 361 (I) N = 482 (C)	Linkage to care any time	43%	33%		Y
						AHR (95% CI)	1.56 (1.24, 1.96)			
Brownrigg et al. (2017) [33]	HCP	Training of nurses in HIV counseling and referrals and implementation of nurse-based counseling and referral system 08/29/2012–08/29/2013	Standard of care: offering confirmatory serology HIV test, providing post-test counselling, and suggesting the client see an HIV physician/clinic by offering a referral 01/01/2011 –12/31/2011	MSM newly diagnosed with HIV in two clinics in Vancouver, Canada	N = 108 (T) N = 51 (I) N = 57 (C)	3-month linkage to care	88%	61%		Y
						Time to linkage to care (median days)	14.0 days [IQR 3–193 days]	21.5 days [IQR 1–75 days]		N
Gordon et al. (2015) [31]	S	New York State 2010 HIV Testing Law mandating that provider (or representative) ordering HIV diagnostic testing, arrange follow-up medical care with an HIV provider 01/01/2011–01/01/2012	Diagnosing provider makes referral without law in place 1/1/2007–12/31/2011	PWH (age 13 and older) in New York state, USA and not perinatally infected	N = 23,302 (T) N = 6850 (I) N = 16,452 (C)	3-month linkage to care	83%	75%		
						Crude OR (95% CI)	1.64 (1.53, 1.76)			
						AOR (95% CI)	1.10 (0.98, 1.24)			
Bocour et al. (2013) [34]	CM	New York City (NYC) Department of Health use of a Field Services Unit (FSU) to provide active linkage to medical care through health system navigation through HIV partner notification services	No HIV partner notification and linkage to care services	Individuals at least age 13 years old newly diagnosed with HIV infection between 2007 and 2011 and reported to the NYC Health Department by 03/31/2013	N = 10,095 (T) N = 4108 (I) N = 5987 (C)	3-month linkage to care	79%	66%		p < 0.0001
						APR (95% CI)	1.10 (1.08, 1.12)			
*Non-randomized experimental study*										
Bendetson et al. (2017) [35]	MC	Licensed clinical social worker with experience in crisis counseling delivers client-centered, resiliency-based counseling and care coordination	None	PWH, newly diagnosed at the Los Angeles LGBT Center, USA	N = 389 (T) N = 118 (I) N = 274 (T) N = 135 (I) N = 139 (C)	3-month linkage to care	94%			
						Time to linkage to care (mean days)	25.5 [SD ± 14.5]			
*Randomized controlled trials*										
Neduzhko et al. (2020) [36]	MC	6-session nurse-delivered case management and text messaging reminders	Referrals to government AIDS treatment centers. No case management nor text messaging reminders	PWH diagnosed in the past 12 months and not yet linked to care. Recruited from one of nine clinics in Ukraine		3-month linkage to care	84%	34%		
						ARR of linkage to care within 3 months (95% CI)	2.45 (1.72, 3.47)			Y
Kenya et al. (2016) [37]	CHW	Community health workers provide care coordination and accompaniment after reactive home-based test	Participant must request community health worker after reactive home-based test	African Americans with newly diagnosed HIV, ages 18–60, Miami, Florida, USA	N = 2 (I) N = 3 (C)	6-week linkage to care	100%	0%		
*Cohort study*										
Christopoulos et al. (2011) [38]	CM	HIV clinic case manager (nurse or social worker) embedded in emergency department for care coordination of new HIV diagnoses	None	PWH newly diagnosed in emergency department through HIV testing program in San Francisco, USA	N = 49	Linkage to care any time (95% CI)	94% (83.1%, 98.7%)			
						7-day linkage to care	73%			
						30-day linkage to care	84%			
						90-day linkage to care	90%			
Miller et al. (2019) [39]	MC	Case management and policy/structural changes collaborating with local departments of health	None	Youth ages 12–24 newly-diagnosed PWH who were referred to each of the eight Adolescent Medical Trials Unit in the USA and not linked to care	N = 2142	42-day linkage to care	70%			
Pitasi et al. (2020) [40]	CM	Case management and health navigation	None	Cis-gender MSM and transgender women with previously undiagnosed HIV infection (at least 50% of whom would be Black or Hispanic/Latino) in the USA	N = 44 (I1) N = 17 (I2) N = 2 (I3)	3-month or 104 day linkage to care	91%	82%	100%	N
	Data system	Use of integrated electronic health records to facilitate client intake and appointment scheduling at the time of positive test				Time to linkage (median days)	5	14	6	N
	HCP	Partnerships with clinical providers to facilitate referrals to care								
Rodriguez et al. (2019) [41]	MC	Direct immediate referral from Department of Health HIV testing facility to bilingual (English and Spanish) case managers and HIV clinic. Clinic reimbursement for first 30 days of medical management and medication	None	PWH newly diagnosed within Florida Department of Health clinics, USA	N = 41	Same-day linkage to care	73%			
						7-day linkage to care	20%			
Seña et al. (2017) [42]	CM	Field service interventionists from state public health system to provide care coordination	None	PWH newly diagnosed in 10 regional health care networks in North Carolina, USA	N = 299	3-month linkage to care from referral	63%			
						1-month linkage to care	29%			
						3-month linkage to care from diagnosis	60%			
Willis et al. (2013) [43]	CM	Clinics funded through District of Columbia Department of Health-provided HIV medical case management service	Clinics without funding for medical case management service	Newly diagnosed PWH identified from the District of Columbia Department of Health surveillance system	N = 817 N = 60 (I) N = 152 (C)	3-month linkage to care AOR (95% CI) 6-month linkage to care AOR (95% CI)	72.4% 0.47 (0.19, 1.15) 80.0% 0.57 (0.23, 1.43)	80.4% 85.1%		p = 0.0082 p = 0.061

*CHW* community health worker, *CM* case management, *HCP* health care provider, *S* structural, *MC* multi-component, *PWH* people with HIV, *MSM*  men who have sex with men, *T* total, *I*  intervention, *C* comparator, *AHR* adjusted hazard ratio, *ARR* adjusted relative risk, *AOR*  adjusted odds ratio, *IQR* interquartile range, *OR* odds ratio, *APR* adjusted prevalence ratio, *CI * confidence interval

Most studies evaluated a CM strategy that involved care coordination, health system navigation, and counseling. Four studies used CM/care coordination alone. One study specifically employed CHWs. Five studies were multiple component interventions that integrated CM/care coordination with either data-to-care [32]; text messaging reminders [36]; collaborations with local health departments to improve tracking and follow-up [39]; or bilingual case managers in department of health HIV testing facilities along with clinic reimbursement for initial HIV medical management [41]. One health care provider (HCP) study represented a nurse-led intervention to deliver HIV counseling and care coordination [33] and one study was structural, specifically legal policy mandating linkage to care [31].

Most studies were in the United States (n = 11), with the others in Canada (n = 1) [33] and Ukraine (n = 1) [36]. Three of the 13 studies specifically focused on sexual and gender minority populations [33, 35, 40]. Eligible populations in two studies specifically focused their intervention on racial/ethnic minorities: Kenya et al. [37] in African Americans in Miami, Florida and Pitasi et al. [40] focused enrollment of at least 50% of the sample from Blacks/African Americans or Hispanic/Latinos. Other studies had at least one-half of the enrolled population as Black/African American or Hispanic/Latino [31, 32, 39, 41, 42]. Sample sizes ranged from 5 to 23,302 participants with a new diagnosis of HIV infection.

### Risk of Bias Assessment

**Fig. 2:**
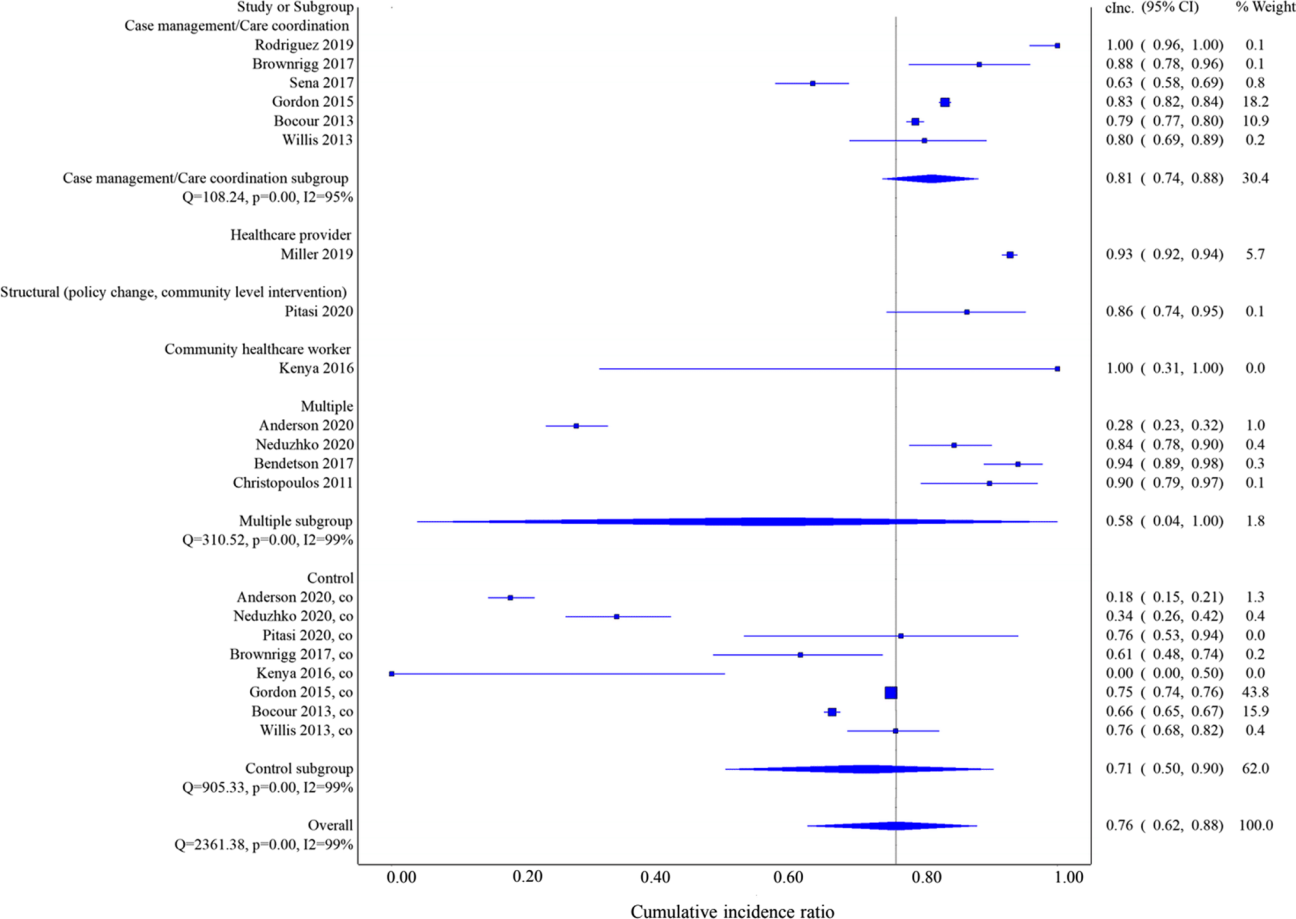
Cumulative incidence of 3-month linkage to care in individual studies, by intervention sub-types compared with controls

Among the 11 non-randomized studies, serious overall risk of bias was observed in seven studies and moderate overall risk of bias was detected in four studies (Online Appendix, Fig. 1). Of the two randomized trials, high risk of bias was detected in one study and low risk of bias in one study (Online Appendix, Fig. 2).

### Meta-analysis

#### Linkage to Care Cumulative Incidence

The overall cumulative incidence of 3-month linkage to care was 0.76 (95% CI 0.62, 0.88) (Fig. 2). When examined by group, the cumulative incidence of linkage to care was 0.82 (95% CI 0.68, 0.94) in the intervention group, with significant heterogeneity (*I*^2^ = 99%, *Q* test *p* value < 0.001, data not shown), and in the control group 0.71 (95% CI 0.50, 0.90), with significant heterogeneity (*I*^2^ = 99%, *Q* test *p* value < 0.001, Fig. 2). Three-month cumulative incidence by intervention type was in CM interventions 0.81 (95% CI 0.74, 0.88), HCP 0.93 (95% CI 0.92, 0.94), structural 0.86 (95% CI 0.74, 0.95), CHW 1.00 (95% CI 0.31, 1.00), and multiple component interventions 0.58 (95% CI 0.04, 1.00). Subgroup analysis by the type of intervention still revealed significant heterogeneity. No significant difference by year of the study or the study design were detected (data not shown).

#### Linkage to Care Cumulative Incidence Ratio

**Fig. 3:**
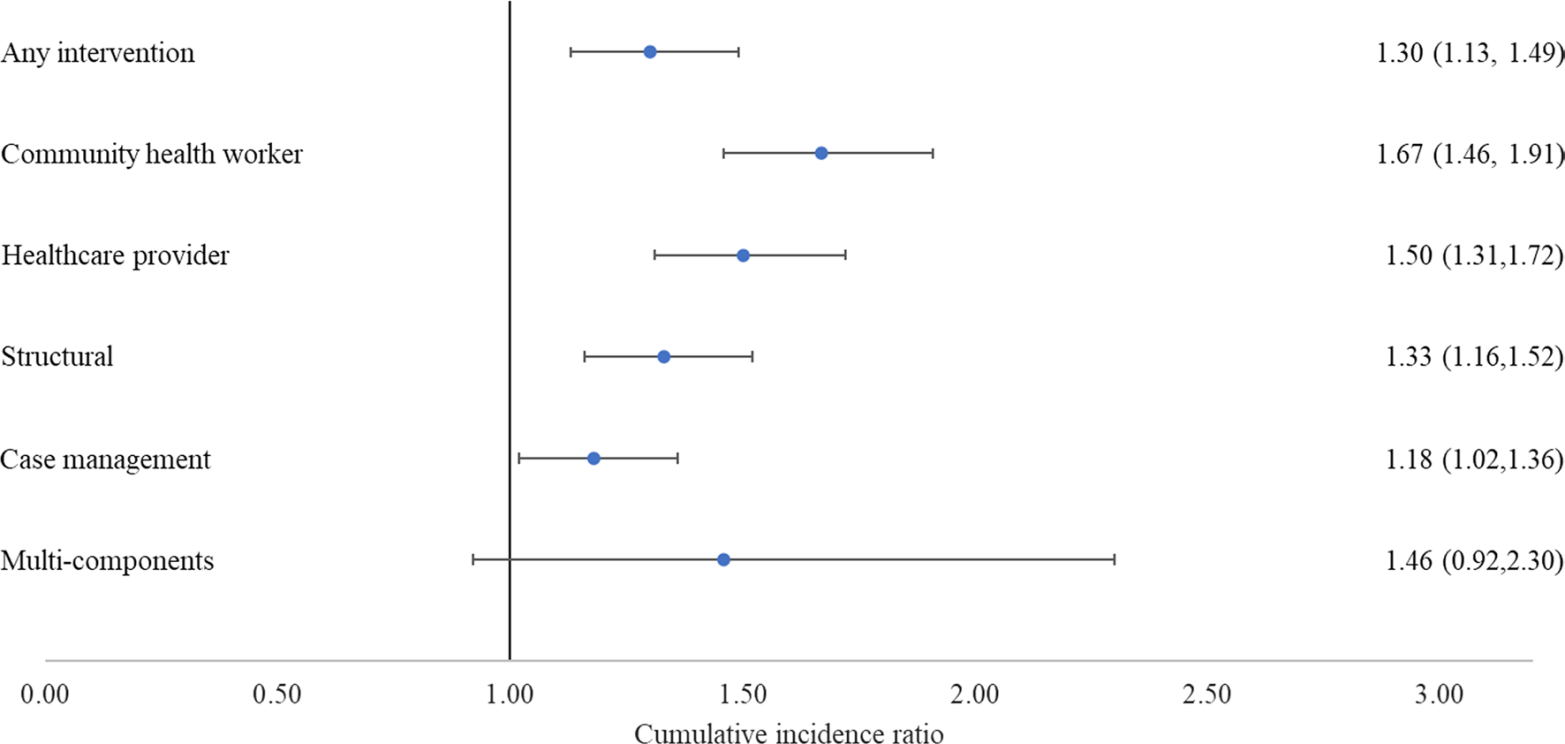
Cumulative incidence ratio of 3-month linkage to care, overall and by intervention type not restricted to 2-arm studies

The cIR of linkage to care in intervention compared with control conditions was 1.30 (95% CI 1.13, 1.49, Fig. 3). The cIR of linkage to care in the CHW intervention was 1.67 (95% 1.46, 1.91), in HCP was 1.50 (95% CI 1.31, 1.72), in the structural intervention was 1.33 (95% CI 1.16, 1.52), and in CM was 1.18 (95% CI 1.02, 1.36). We did not detect a significant increase in 3-month linkage to care in association with multiple component interventions, which was 1.46 (95% CI 0.92, 2.30).

**Fig. 4:**
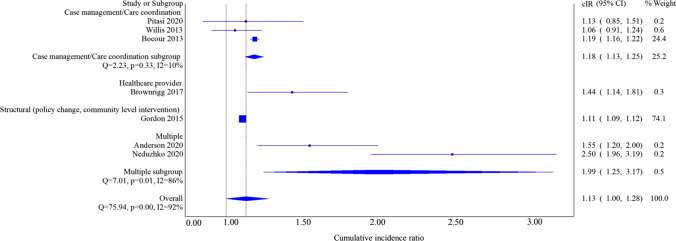
Cumulative incidence ratio of 3-month linkage to care, overall and by intervention type restricted to 2-arm studies

When the analysis was restricted to 2-arm studies, the overall pooled 3-month cIR was 1.13 (95% CI 1.00, 1.28). We detected significant heterogeneity (*I*^2^ = 92%, *Q* test *p* value < 0.001, Fig. 4). The likelihood of 3-month linkage to care in HCP was 1.44 (95% CI 1.14, 1.81), structural intervention was 1.11 (95% CI 1.09, 1.12), CM was 1.18 (95% CI 1.13, 1.25), and multiple component interventions was 1.99 (95% CI 1.25, 3.17). Subgroup analysis by intervention type, revealed no heterogeneity in the CM/case coordination studies (*I*^*2*^ = *10%, Q test p value* = *0.33*), however we detected significant heterogeneity in the multiple component interventions (*I*^2^ = 86%, *Q* test *p* value = 0.01, Fig. 4).

### Risk of Reporting Biases (Publication Bias)

Doi, plot, and LFK index detected no publication bias (Online Appendix, Fig. 3). Specifically, the LFK index was − 0.16, indicating no publication bias.

## Discussion

At least one-fifth of PWH in North America and Europe do not successfully link to HIV care for ART, which compromises their individual health, increases the risk of HIV transmission, and undermines national and global aspirations for ending the HIV epidemic. We conducted a systematic review and meta-analysis in the contemporary ART era (2010 to 2021) and identified 13 published studies focusing specifically on interventions to improve linkage to HIV care in the US, Canada, and Ukraine. Interventions increased the rate of linkage to care at three months by 30% compared with control conditions and by 13% when restricted to 2-arm studies, across a broad array of intervention types.

Most studies evaluated an intervention that had CM and care coordination as a core component of the intervention and our findings underscore the importance of these components in linkage to care. Prior in-depth qualitative research also demonstrates that linkage to care is a multi-component process that includes identifying and contacting individuals newly diagnosed with HIV, evaluating and addressing individual needs and barriers to care, and facilitating initial engagement to HIV primary care [44]. Furthermore, these studies have uncovered that barriers to linkage to care include provider knowledge on the referral process, communication challenges between different service testing sites and clinics, and lack of co-location of services. Problems are exacerbated for clients with co-occurring needs and additional barriers including substance use disorders, mental health problems, and socioeconomic disadvantage [45].

One intervention that utilized CHWs for linkage to care was a non-randomized study design with a small sample size but found a 70% increase in linkage to care compared with the control condition [37]. Task shifting HIV clinical efforts to CHWs has an established role in disseminating HIV care into community settings in a cost-effective manner [46]. Community and lay health workers, often drawn from the local community, can deliver public health interventions responsive to local needs with the necessary cultural and linguistic aptitude [47, 48]. No studies specifically focused on people who inject drugs, yet they are at increased risk of attrition from HIV care and may benefit from CHWs to support integration of clinical and non-clinical services in the community setting.

A legal intervention to mandate linkage to care by the HIV testing provider significantly increased linkage to care in the primary analysis by 33% and when the analysis was restricted to 2-arm studies by 11% compared with control conditions [31]. This state-wide initiative in New York included a sample size of nearly 23,000 individuals [31]. A New York State 2010 HIV testing law mandates that the diagnosing provider is responsible for providing a referral to HIV primary care. In this study, medical care was inferred by the presence of HIV laboratory data (CD4 count and HIV viral load report). There were improvements in state-wide collection of HIV testing data over time. Therefore, it was not possible to determine if the increase in linkage to care related to institution of the legal mandate on providers to refer into HIV care or improved laboratory reporting. To facilitate providers meeting their reporting requirements, the New York State Department of Health created an internet-based portal [49]. This data sharing, or “data-to-care,” platform enables departments of health to identify and contact individuals newly diagnosed with HIV and allows for communication between departments of health and HCPs. To our knowledge, New York is the only state with a legal mandate on the diagnosing provider for linkage to care, yet in 48 states and Puerto Rico there are laws mandating HIV laboratory reporting to the state to facilitate care [50].

The COVID-19 pandemic and war in Ukraine that has displaced at least 12 million people, highlight the more recent crises challenging HIV prevention and care [16]. These realities underscore the importance of implementing evidence-based linkage to care interventions such as the nurse-delivered CM intervention for people with HIV in Ukraine evaluated in the Modified Antiretroviral Treatment Access Study (MARTAS) [36].

Our analysis has several limitations. Only two of the studies we found were randomized controlled trials, the standard experimental method for testing intervention effectiveness. This review highlights the heterogeneity in study outcomes on linkage to care, which limits comparability with current recommendations to link patients as soon as possible and ideally within one month of diagnosis [5]. We focused eligibility criteria on studies targeting linkage to care, rather than HIV testing, though in doing so we may have missed studies where the modality of HIV testing facilitated linkage to care. Only one study, from Ukraine, met inclusion criteria for Europe and may not be generalizable to other countries and regions within Europe. In addition, we discovered significant heterogeneity that subgroup analysis did not explain, except for the CM/care coordination studies. Heterogeneity could be attributed to methodological differences, wide variability in the intervention types (even the types that belong to the same subgroup), and variability in the study populations and sample sizes. Additionally, due to the small number of studies included in the CM/care coordination intervention subgroup, the observed low I^2^ statistic might be biased, leading to underestimation of heterogeneity [51]. We also detected high risk of bias due to the observational nature of the studies.

This study also has strengths, including capturing existing studies with diverse racial/ethnic study composition, which is essential since Black and Latinx populations are disproportionately impacted by the HIV epidemic in the US. Furthermore, the studies included occurred in geographic “hotspots” with elevated HIV seroprevalence such as the US Southeast region; Los Angeles, California; Vancouver, Canada; and Ukraine with interventions that could be implemented in similar settings.

In summary, in this systematic review and meta-analysis of HIV linkage to care interventions in the US, Canada, and Ukraine, we found that multiple interventions improve linkage, including CM either alone or as part of multi-component interventions. Additional measures to improve linkage to care may include integration of CHWs into HIV care teams, engaging departments of health to assist in identification and tracking of individuals newly diagnosed with HIV, and enhanced accountability of diagnosing providers in the referral process. Characterization and reporting of descriptive characteristics of the study populations (e.g. age, substance use disorder, and mental health history) as well as the reporting of outcome data such as odds and incidence ratios, will improve future analyses and the ability to detect confounding on linkage to care. If goals to markedly decrease, or end, incident cases of HIV in the US, Canada, and Europe are to be achieved, better interventions for linkage to HIV care are needed.

### Registration and Protocol

We have registered this study in the International Prospective Register of Systematic Reviews (PROSPERO) [52]. The protocol registration number is CRD42021235610. The registration includes a protocol, which is available online. No protocol amendment has been done.

### Supplementary Information

Below is the link to the electronic supplementary material.

Supplementary file1 (DOCX 574 KB)

## Data Availability

All extracted data are provided in the tables; therefore there are no additional data are available.

## Code Availability

Codes are available from the authors upon request.
