# Supplementary material for: Systematic Review and Meta-analysis of Linkage to HIV Care Interventions in the United States, Canada, and Ukraine (2010–2021)
Source: AIDS Behav. 2023 Jul 31;27(12):4070–83. doi: 10.1007/s10461-023-04121-0 (PMC10598185; doi:10.1007/s10461-023-04121-0)
Supplement: Supplementary file 1 — Supplementary file1 (DOCX 574 KB) [file 10461_2023_4121_MOESM1_ESM.docx]

**Systematic review and meta-analysis of linkage to HIV care interventions in the United States, Canada, and Ukraine (2010-2021)**

**Supplementary Materials**

**Appendix**

Search Strategy

**Appendix Table I, Full Search Strategy for PubMed (MEDLINE) Searched on 1/1/21**

| #1 | ("Acquired Immunodeficiency Syndrome"[MeSH] OR "HIV"[MeSH] OR human immunodeficiency virus[tiab] OR acquired immunodeficiency syndrome[tiab] OR HIV[tiab] OR AIDS[tiab] OR "HIV Infections"[MeSH]) |
| --- | --- |
| #2 | (("Program evaluation"[MeSH] OR intervention* to increase[tiab] OR intervention trial*[tiab] OR "evaluation studies"[publication type] OR intervention* to improve[tiab] OR Intervention stud*[tiab] OR Strateg* to increase[tiab] OR strateg* to improve[tiab] OR Methodolog* to improve[tiab] OR Linkage intervention*[tiab] OR HIV-testing-outcomes[tiab])) |
| #3 | (link to care[tiab] OR linkage to care[tiab] OR linkages to care[tiab] OR care linkage*[tiab] OR treatment initiati*[tiab] OR linkage to medical care[tiab] OR linkage-to-HIV-medical-care[tiab] OR linking and retaining[tiab] OR therapy initiati*[tiab] OR care initiati*[tiab] OR start treatment*[tiab] OR starting treatment*[tiab] OR start therap*[tiab] OR starting therap*[tiab] OR linkage to[tiab] OR (link[ti] AND HIV[ti])) |
| #4 | #1 and #2 AND #3 |

**Appendix Table II, Full Search Strategy for Embase.com Searched on 1/1/21**

| #1 | Acquired Immunodeficiency Syndrome'/exp OR 'Human immunodeficiency virus'/exp OR 'human immunodeficiency virus':ti,ab OR 'acquired immunodeficiency syndrome':ti,ab OR HIV:ti,ab OR AIDS:ti,ab |
| --- | --- |
| #2 | 'intervention* to increase':ti,ab OR 'intervention trial*':ti,ab OR 'intervention study'/exp OR 'evaluation study'/exp OR 'intervention* to improve':ti,ab OR 'Intervention stud*':ti,ab OR 'Strateg* to increase':ti,ab OR 'strateg* to improve':ti,ab OR 'Methodolog* to improve':ti,ab OR 'Linkage intervention*':ti,ab OR 'HIV testing outcomes':ti,ab |
| #3 | linkage to care'/exp OR 'treatment initiation'/exp OR 'link to care':ti,ab OR 'linkage to care':ti,ab OR 'linkages to care':ti,ab OR 'care linkage*':ti,ab OR 'treatment initiati*':ti,ab OR 'linkage to medical care':ti,ab OR 'linkage to HIV medical care':ti,ab OR 'therapy initiati*':ti,ab OR 'care initiati*':ti,ab OR 'start treatment*':ti,ab OR 'starting treatment*':ti,ab OR 'start therap*':ti,ab OR 'starting therap*':ti,ab OR 'linkage to':ti,ab OR 'Linking and retaining':ti,ab OR (link:ti AND HIV:ti) |
| #4 | #1 and #2 AND #3 |

**Appendix Table III, Full Search Strategy for Cochrane Searched on 1/1/21**

| #1 | (human immunodeficiency virus or acquired immunodeficiency syndrome or HIV or AIDS).ti,ab. |
| --- | --- |
| #2 | (intervention* to increase or intervention trial*).ti,ab. or evaluation studies.pt. or intervention* to improve.ti,ab. or Intervention stud*.ti,ab. or Strateg* to increase.ti,ab. or strateg* to improve.ti,ab. or Methodolog* to improve.ti,ab. or Linkage intervention*.ti,ab. |
| #3 | ((link to care).ti,ab. OR (linkage to care).ti,ab. OR (linkages to care).ti,ab. OR (care linkage*).ti,ab. OR (treatment initiati*).ti,ab. OR (linkage to medical care).ti,ab. OR (linkage to HIV medical care).ti,ab. OR (linking and retaining).ti,ab. OR (therapy initiati*).ti,ab. OR (care initiati*).ti,ab. OR (start treatment*).ti,ab. OR (starting treatment*).ti,ab. OR (start therap*).ti,ab. OR (starting therap*).ti,ab. OR (linkage to).ti,ab. OR (link.ti. AND HIV.ti.)) |
| #4 | #1 and #2 AND #3 |

**Appendix Table IV, Full Search Strategy for Web of Science Searched on 1/1/21**

| #1 | TS=("Acquired Immunodeficiency Syndrome" OR "human immunodeficiency virus" OR HIV OR AIDS) |
| --- | --- |
| #2 | TS=("intervention* to increase" OR "intervention trial*" OR "intervention study" OR "evaluation study" OR "intervention* to improve" OR "Intervention stud*" OR "Strateg* to increase" OR "strateg* to improve" OR "Methodolog* to improve" OR "Linkage intervention*" OR "HIV testing outcomes") |
| #3 | TS=("linkage to care" OR "treatment initiation" OR "link to care" OR "linkage to care" OR "linkages to care" OR "care linkage*" OR "treatment initiati*" OR "linkage to medical care" OR "linkage to HIV medical care" OR "therapy initiati*" OR "care initiati*" OR "start treatment*" OR "starting treatment*" OR "start therap*" OR "starting therap*" OR "linkage to" OR "Linking AND retaining" OR (link AND HIV)) |
| #4 | #1 and #2 AND #3 |

**Appendix Table V, Full Search Strategy for CINAHL Searched on 1/1/21**

| #1 | ((MH "Acquired Immunodeficiency Syndrome+") OR (MH "HIV+") OR (TI "human immunodeficiency virus" OR AB "human immunodeficiency virus") OR (TI "acquired immunodeficiency syndrome" OR AB "acquired immunodeficiency syndrome") OR (TI HIV OR AB HIV) OR (TI AIDS OR AB AIDS)) |
| --- | --- |
| #2 | (((MH "Program evaluation+") OR (MH "Intervention Trials+") OR (TI "intervention* to increase" OR AB "intervention* to increase") OR (TI "intervention trial*" OR AB "intervention trial*") OR (TI "evaluation stud*" OR AB "evaluation stud*") OR (TI "intervention* to improve" OR AB "intervention* to improve") OR (TI "Intervention stud*" OR AB "Intervention stud*") OR (TI "Strateg* to increase" OR AB "Strateg* to increase") OR (TI "strateg* to improve" OR AB "strateg* to improve") OR (TI "Methodolog* to improve" OR AB "Methodolog* to improve") OR (TI "Linkage intervention*" OR AB "Linkage intervention*") OR (TI "HIV testing outcomes" OR AB "HIV testing outcomes"))) |
| #3 | ((TI "link to care" OR AB "link to care") OR (TI "linkage to care" OR AB "linkage to care") OR (TI "linkages to care" OR AB "linkages to care") OR (TI "care linkage*" OR AB "care linkage*") OR (TI "treatment initiati*" OR AB "treatment initiati*") OR (TI "linkage to medical care" OR AB "linkage to medical care") OR (TI "linkage to HIV medical care" OR AB "linkage to HIV medical care") OR (TI "linking and retaining") OR (AB "linking and retaining") OR (TI "therapy initiati*" OR AB "therapy initiati*") OR (TI "care initiati*" OR AB "care initiati*") OR (TI "start treatment*" OR AB "start treatment*") OR (TI "starting treatment*" OR AB "starting treatment*") OR (TI "start therap*" OR AB "start therap*") OR (TI "starting therap*" OR AB "starting therap*") OR (TI "linkage to" OR AB "linkage to") OR (TI link AND TI HIV)) |
| #4 | #1 and #2 AND #3 |

CINAHL= Cumulative Index to Nursing and Allied Health Literature

Additional Methods

*Risk of bias assessment*

We used two risk of bias assessment tools. For randomized trials we used the revised Cochrane “Risk of bias” tool for randomized trials (RoB 2.0) (19). For non-randomized studies we used the “Risk of bias” tool called ROBINS-I (20). RoB 2.0 addresses five domains: 1) bias due to the randomization process, 2) bias due to deviation from the intervention, 3) bias due to missing outcome, 4) bias in the measurement of the outcome, and 5) bias in selection of the reported results. Similarly, ROBINS-I assesses seven domains; three of the domains are not assessed by RoB 2.0, which are 1) bias due to confounding, 2) bias due to selection of participants, and 3) bias in classification of intervention, and four of the domains overlap with RoB 2.0 i.e., 4) bias due to departure from the intervention, 5) bias due to missing data, 6) bias in the measurement of the intervention, and 7) bias in selection of reported results (18). We derived an overall “risk of bias summary,” which classified each study as “low, some concern, and high” risk of bias. Two reviewers (JVM and DS) independently evaluated the studies. In the presence of discrepancies, a third reviewer (FMS or JHL) resolved the disagreements. We used Risk-of-bias VISualization (robvis) to create risk-of-bias plots (21).

*Synthesis methods*

*Outcome*

Linkage to care outcomes were not reported in a standardized fashion. Therefore, because most studies reported linkage to care within three months, we used the reported cumulative proportions, with the time frame, or the number of events and the time frame to estimate linkage rate per month, and subsequently estimated the 3-month linkage to care probability (cumulative incidence) from the following equation:

$$Linkage rate(r)=\frac{Number of events}{Time}$$

$$Linkage rate (r)= \frac{-ln\left( 1-cumulative proportion \right)}{Time}$$

$$3-month linkage probability=1-{exp}^{\left( -rt \right)}$$

Therefore, the main study outcome was 3-month linkage to care cumulative incidence.

We planned to use the cumulative incidence ratios (cIR) of the outcome reported by each study. However, studies often did not provide measures of cIR, but provided other estimates such as time to linkage, or the frequencies of the outcome in the intervention and the comparator groups. Therefore, we estimated a pooled cIR using two approaches; 1) calculating the estimate from all studies (single-arm and 2-arm studies) using a meta-regression approach, and 2) restricting to studies that included a comparator group. In the first approach, to be able to use single-arm studies, we used a meta-regression approach, such that 2-arm studies contributed control and intervention data and single-arm studies contributed intervention data. Subsequently, cumulative incidence was compared in the intervention to the control, and also intervention subtypes were compared to control, accounting for the intra-study correlation. In the second approach, we examined the outcome of linkage to care only including 2-arm studies such that the intervention and the comparator groups were compared within each study.

*Reporting bias assessment (publication bias)*

We used Doi plot and the Luis Furuya-Kanamori (LFK) index, a quantitative indicator of Doi plot asymmetry, for the detection of publication bias (30). Doi plots were selected to aid interpretability in meta-analyses. We also used the LFK index because of its higher power to detect asymmetry compared to other methods such as Egger’s regression (30). Based on the LFK index, values that are <+ 1 indicate no asymmetry, values ranging from +1 and +2 indicate minor asymmetry, and values >+2 indicate major asymmetry.

Supplementary Data

**Figure 1**: Risk of bias assessment of non-randomized studies.

**Figure 2**: Risk of bias assessment of randomized studies.

**Figure 3**: Publication bias (doi plot) in a systematic review and meta-analysis of linkage to care in US, Canada, and Ukraine.

**Appendix Figure 1**


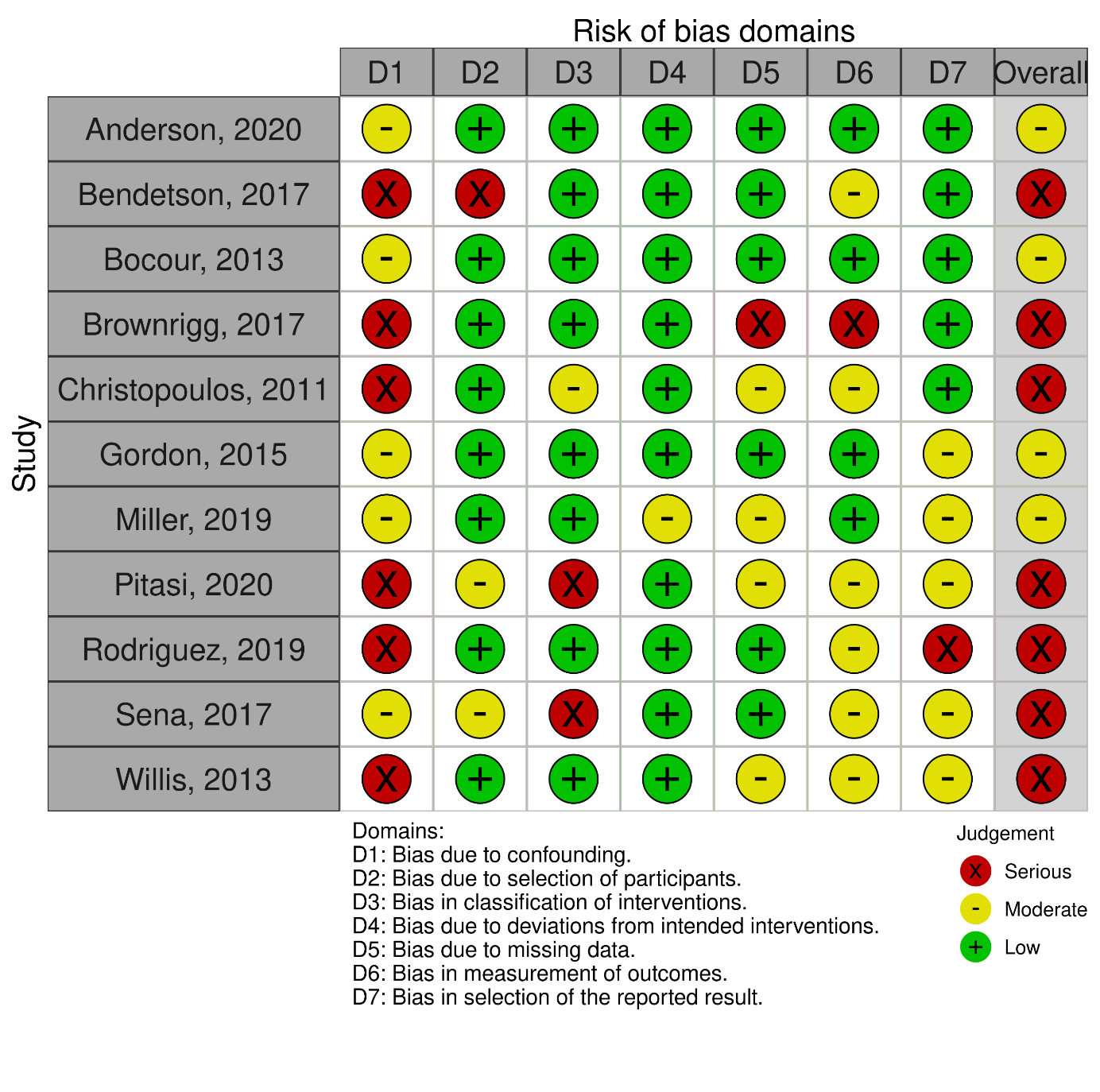


**Appendix Figure 2**


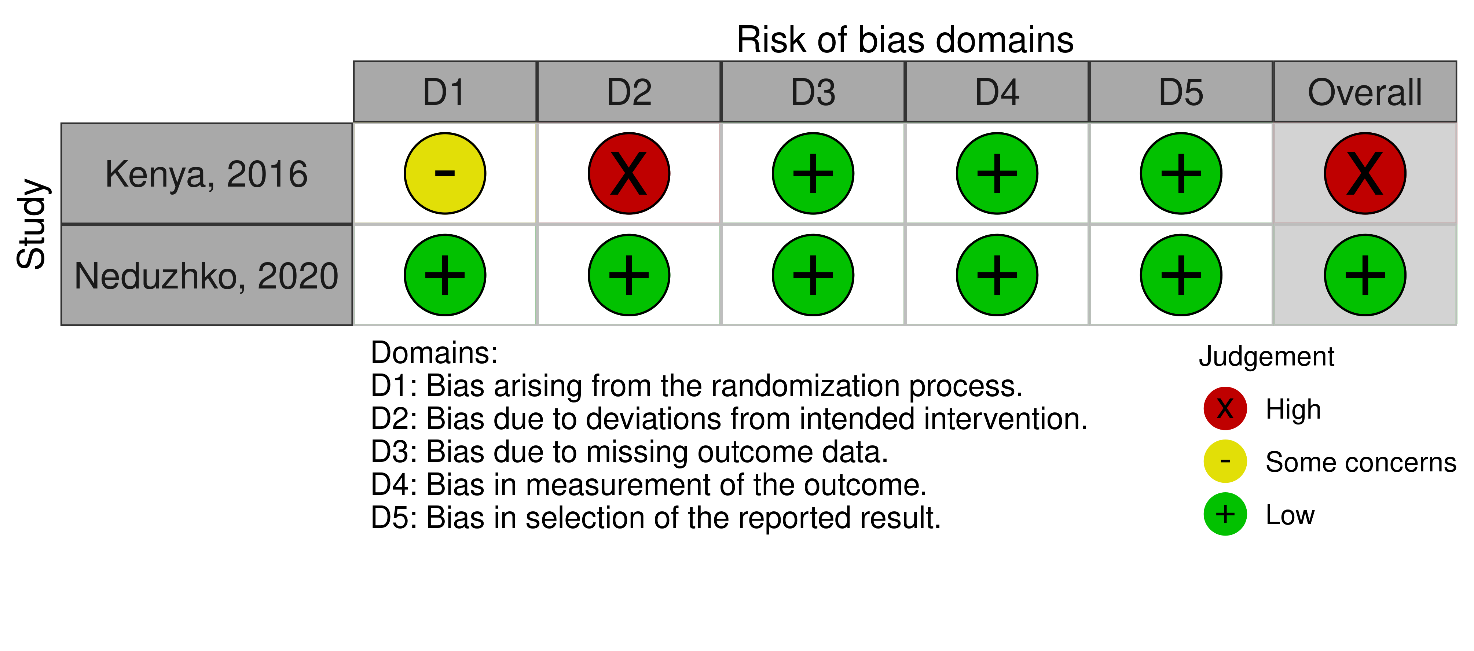


**Appendix Figure 3**

Double arcsine cumulative incidence

Z-score

LFK Index; -0.16 (No asymmetry)
